# Physical Activity During Pregnancy, Dietary Adequacy, and Energy-Dense, Nutrient-Poor Food and Beverage Intake: Associations with Preterm Birth

**DOI:** 10.3390/nu18122030

**Published:** 2026-06-22

**Authors:** Oana Liliana Atomei, Petronela Vicoveanu, Dragos Vicoveanu, Monica Tarcea

**Affiliations:** 1 Doctoral School, George Emil Palade University of Medicine, Pharmacy, Science, and Technology of Targu Mures, 540139 Targu Mures, Romania; 2 Department of Biological and Morphofunctional Sciences, Faculty of Medicine and Biological Sciences, Stefan cel Mare University of Suceava, 720229 Suceava, Romania; 3 Department of Medical-Surgical and Complementary Sciences, Faculty of Medicine and Biological Sciences, Stefan cel Mare University of Suceava, 720229 Suceava, Romania; 4 Obstetrics and Gynecology Department, Emergency County Clinical Hospital St. Ioan cel Nou, 720224 Suceava, Romania; 5 Department of Computers, Electronics and Automation, Faculty of Electrical Engineering and Computer Science, Stefan cel Mare University of Suceava, 720225 Suceava, Romania; dragos.vicoveanu@usm.ro; 6 Department of Community Nutrition and Food Safety, George Emil Palade University of Medicine, Pharmacy, Science, and Technology of Targu Mures, 540139 Targu Mures, Romania; monica.tarcea@umfst.ro

**Keywords:** pregnancy, physical activity, dietary adequacy, energy-dense, nutrient-poor foods, fruit and vegetable intake, sugar-sweetened beverages, preterm birth, Pregnancy Physical Activity Questionnaire

## Abstract

**Background/Objectives:** Maternal nutrition and physical activity are modifiable behaviours relevant to pregnancy outcomes, but higher activity may coexist with both favourable and unfavourable dietary patterns. This study examined associations between pregnancy physical activity, individualised fruit–vegetable adequacy, energy-dense, nutrient-poor (EDNP) food and beverage intake, and preterm birth. **Methods:** This cross-sectional study included 1048 postpartum women with singleton live births recruited consecutively at a tertiary maternity hospital in Romania. Physical activity during the last three months of pregnancy was assessed using the Pregnancy Physical Activity Questionnaire and categorised into quartiles of total MET-hours/week. Dietary intake was assessed using an adapted food frequency questionnaire. Fruit–vegetable adequacy was evaluated against individualised recommendations, and EDNP intake was summarised using a composite score derived from fast food, sweets, chocolate, and sugar-sweetened beverages. Preterm birth was defined as delivery before 37 completed weeks of gestation. **Results:** Preterm birth occurred in 118 cases (11.3%). Higher physical activity categories showed greater fruit–vegetable intake and adequacy, but also higher EDNP intake. After adjustment for maternal age, pregestational BMI, parity, education, and income, physical activity category remained associated with all modelled dietary outcomes. Category 4 had higher odds of fruit–vegetable adequacy than category 1 (OR 2.24, 95% CI 1.55–3.24). In diet-informed models, category 3 had the lowest odds of preterm birth (OR 0.38, 95% CI 0.21–0.68). **Conclusions:** Total physical activity during pregnancy was associated with a complex dietary profile rather than a uniformly favourable lifestyle pattern. The lowest odds of preterm birth were observed in the third activity category, suggesting a non-linear association.

## 1. Introduction

Preterm birth remains a major global public health concern and a leading contributor to neonatal morbidity and mortality. According to the World Health Organization, an estimated 13.4 million infants were born preterm in 2020, corresponding to more than one in ten live births worldwide [1]. Although its etiology is heterogeneous, preterm birth is increasingly understood as the result of interactions between maternal biological vulnerability, metabolic status, lifestyle behaviours, and environmental context. Within this framework, maternal nutrition and physical activity represent potentially modifiable determinants of maternal–fetal health, but their joint interpretation remains complex.

Maternal diet during pregnancy contributes to fetal growth, placental function, maternal metabolic adaptation, and inflammatory balance [2,3]. Evidence from observational studies and systematic reviews suggests that dietary patterns rich in vegetables, fruits, whole grains, fish, and minimally processed foods are generally associated with more favourable pregnancy outcomes, whereas dietary profiles characterised by higher intake of ultra-processed foods, sugar-sweetened beverages, and energy-dense, nutrient-poor (EDNP) foods may be associated with less favourable maternal or perinatal outcomes [3,4,5,6]. However, associations between individual dietary components and preterm birth have been less consistent than those observed for broader dietary patterns, suggesting that isolated food groups may insufficiently capture the nutritional and behavioural complexity of pregnancy diet [4].

Fruit and vegetable intake is commonly used as an indicator of dietary adequacy because these foods provide fibre, antioxidants, folate, potassium, and other micronutrients relevant to maternal and placental health. Nevertheless, the interpretation of fruit–vegetable adequacy during pregnancy depends not only on absolute intake but also on maternal energy requirements, which vary according to age, body size, gestational energy needs, and physical activity level. Individualised adequacy indicators may therefore provide a more clinically meaningful assessment than uniform intake thresholds alone.

EDNP foods and beverages represent a pragmatic marker of less favourable dietary behaviour. In pregnancy, frequent consumption of fast-food products, sweets, chocolate, and sugar-sweetened beverages may contribute to excess energy intake, poorer diet quality, metabolic dysregulation, and inflammatory pathways potentially relevant to adverse obstetric outcomes [5,6]. EDNP intake should therefore be interpreted not only as an isolated dietary exposure, but also as part of a broader maternal lifestyle profile that may coexist with other behavioural characteristics, including physical activity.

Physical activity during pregnancy is generally recommended for women without obstetric or medical contraindications. Current guidelines indicate that regular moderate-intensity activity during pregnancy is associated with maternal health benefits and minimal risk for most women [7,8]. Evidence from systematic reviews suggests that prenatal exercise is not associated with increased risk of preterm birth in uncomplicated pregnancies and may improve several maternal and neonatal outcomes [9]. However, physical activity in epidemiological studies is not limited to structured exercise. Pregnancy physical activity questionnaires capture multiple domains, including household, caregiving, transportation, occupational, sports/exercise, and sedentary activities. Thus, total physical activity expressed in MET-hours/week may reflect a heterogeneous construct rather than leisure-time exercise alone [10,11].

This heterogeneity is important when physical activity is examined together with diet. Higher total physical activity may indicate healthier behaviour, but it may also reflect greater daily workload, increased energy requirements, or higher overall food intake. Consequently, more active pregnant women may not necessarily present a uniformly healthier dietary profile. Instead, physical activity may be associated with both greater dietary adequacy and higher consumption of EDNP foods. This possibility is relevant for nutritional epidemiology because it challenges the assumption that lifestyle behaviours cluster in a uniformly favourable or unfavourable direction.

Despite increasing interest in both nutrition and physical activity during pregnancy, few studies have examined whether physical activity categories are simultaneously associated with fruit–vegetable adequacy, EDNP intake, and preterm birth. Such an integrated approach may help clarify whether Pregnancy Physical Activity Questionnaire (PPAQ)-derived physical activity categories identify a broadly favourable lifestyle pattern or a more complex profile in which dietary adequacy and EDNP intake coexist.

This study aimed to examine the associations between maternal physical activity during pregnancy, fruit–vegetable adequacy, EDNP food and beverage intake, and preterm birth in a Romanian sample of postpartum women. The specific objectives were: (1) to compare maternal dietary profiles across physical activity categories; (2) to evaluate whether physical activity categories were associated with fruit–vegetable adequacy and EDNP intake after adjustment for relevant maternal and socioeconomic factors; and (3) to assess the association between physical activity categories and preterm birth, including diet-informed adjustment for EDNP intake and fruit–vegetable adequacy. We hypothesised that physical activity categories would be associated with both dietary adequacy and EDNP intake, and that PPAQ-derived physical activity categories would be associated with the odds of preterm birth after adjustment for relevant maternal and dietary factors. By integrating physical activity, dietary adequacy, EDNP intake, and preterm birth in the same analytical framework, this study was designed to assess whether total pregnancy physical activity identifies a uniformly favourable lifestyle profile or a more complex maternal behavioural pattern.

## 2. Materials and Methods

### 2.1. Study Design and Participants

This cross-sectional study was conducted among postpartum women recruited consecutively between July 2024 and July 2025 at the Emergency County Clinical Hospital St. Ioan cel Nou in Suceava, Romania, a tertiary-level regional maternity hospital.

Eligible participants were women aged ≥18 years with singleton pregnancies who gave birth to live-born neonates during the recruitment period. Both term and preterm births were included. Exclusion criteria were multiple pregnancy, neonatal macrosomia defined as birth weight ≥ 4000 g, maternal diabetes mellitus recorded before or during pregnancy, maternal death during hospitalisation, neonatal death during hospitalisation, refusal to provide written informed consent, or incomplete questionnaire data. The exclusion of neonatal macrosomia was predefined in the study protocol to reduce etiological heterogeneity related to fetal growth extremes and metabolic pathways distinct from those typically implicated in preterm birth.

A total of 1482 mother–newborn medical records were screened for eligibility. After application of the exclusion criteria, 1347 mother–newborn pairs remained eligible. Of these, 1123 women agreed to participate and provided written informed consent. Complete questionnaire and clinical data were available for 1048 mother–newborn pairs included in the final statistical analyses.

### 2.2. Data Collection

Data were obtained from obstetric and neonatal medical records, a self-administered maternal questionnaire completed during postpartum hospitalisation, and the hospital electronic registry used to verify clinical variables.

Maternal variables relevant to the present analysis included age, pregestational weight and height, gestational age at birth, parity, smoking during pregnancy, alcohol use during pregnancy, supplement use, educational level, and household income. Neonatal information relevant to this analysis included birth status according to gestational age and birth weight.

The questionnaire was administered in Romanian using an electronic tablet and was completed once, within 1–5 days after delivery. It included sections on dietary intake during pregnancy, socioeconomic characteristics, physical activity, and selected pregnancy-related behaviours. Because exposure data were collected postpartum, dietary intake was assessed retrospectively for the pregnancy period, whereas physical activity was assessed retrospectively for the last three months of pregnancy.

### 2.3. Physical Activity Assessment

Physical activity during the last three months of pregnancy was assessed using the Pregnancy Physical Activity Questionnaire (PPAQ), which captures multiple domains of activity, including household/caregiving, occupational, sports/exercise, transportation, sedentary, and other activities [10]. Total physical activity was expressed as MET-hours/week.

For each participant, total physical activity was calculated by multiplying the estimated duration of each reported activity by its corresponding metabolic equivalent value and summing across activities. MET values were assigned according to the Compendium of Physical Activities [11].

Total physical activity was categorised into four sample-based categories using quartiles of the MET-hours/week distribution. The four categories were defined as follows: category 1, ≤225.34 MET-hours/week; category 2, >225.34 to ≤341.51 MET-hours/week; category 3, >341.51 to ≤482.62 MET-hours/week; and category 4, >482.62 MET-hours/week. These categories were used as the main exposure variable, with the lowest physical activity category as the reference group in adjusted general linear and logistic regression models. Because the present analysis focused on total PPAQ-derived MET-hours/week rather than intensity-specific activity categories, these sample-based categories should be interpreted as relative exposure levels within the study population rather than as clinical or guideline-based activity thresholds.

### 2.4. Dietary Assessment

Maternal dietary intake during pregnancy was assessed using an adapted food frequency questionnaire derived from selected items of the Norwegian Mother and Child Cohort Study questionnaires [12]. The questionnaire evaluated usual frequency of consumption of selected foods and beverages during pregnancy using predefined frequency categories.

Portion size estimation was supported by a standardised A–D visual portion guide corresponding to 0.25, 0.5, 1, and 2 cups [13]. Reported frequencies were converted into daily frequencies, and estimated daily intakes were calculated as grams/day or millilitres/day using NutriBase Clinical Nutrition Manager v7.18 (CyberSoft, Inc., Phoenix, AZ, USA). For beverages, one glass was defined as 240 mL and one cup as 120 mL.

### 2.5. Fruit–Vegetable Intake and Individualised Adequacy

Total fruit–vegetable intake was calculated as the sum of daily vegetable and fruit intake, expressed in grams/day. Dietary adequacy was assessed using an individualised indicator comparing each participant’s observed fruit–vegetable intake with the recommended intake corresponding to her estimated energy requirement.

Estimated energy requirements were derived according to the Dietary Reference Intakes framework using maternal age, pregestational weight, height, and a fixed low-active physical activity coefficient [14]. Pregnancy-related additional energy needs were incorporated, and recommended fruit and vegetable intakes were assigned according to the Healthy U.S.-Style Dietary Pattern for pregnant or lactating women from the Dietary Guidelines for Americans 2020–2025 [15].

A fixed low-active coefficient was used for all participants in the main adequacy analysis to avoid circularity between the physical activity exposure and the fruit–vegetable adequacy outcome. Fruit–vegetable intake was classified as adequate when observed intake met or exceeded the individualised recommendation. This binary adequacy indicator was subsequently used as the dietary outcome in logistic regression analyses, with inadequate intake serving as the reference category.

### 2.6. Energy-Dense, Nutrient-Poor Dietary Indicators

EDNP intake was operationalised using selected dietary indicators derived from the food frequency questionnaire. Fast-food intake was calculated as the sum of prepared sandwich-type meals, kebab/shaorma, pizza, and French fries intake, expressed in grams/day. In this context, the sandwich item referred to prepared meals listed in the hot-meal section of the questionnaire, rather than to all bread-based meals. Sweets intake included cakes, pies, pancakes, doughnuts, wafers, candy, cereal bars, and ice cream, expressed in grams/day. Sugar-sweetened beverages included cola beverages, other carbonated soft drinks, sweetened non-carbonated beverages, and energy drinks, expressed in millilitres/day.

A composite EDNP dietary score was created from fast-food, sweets, chocolate, and sugar-sweetened beverage intake. Because these components showed right-skewed distributions, each component was first transformed as ln (1 + intake), with intake expressed in grams/day or millilitres/day as appropriate. The transformed components were then standardised as z-scores and summed. The resulting composite score was subsequently re-standardised as a z-score in the analytical sample, so that higher values indicated greater EDNP food and beverage intake. In adjusted models evaluating individual intake outcomes, fast-food intake, sweets intake, sugar-sweetened beverage intake, and total fruit–vegetable intake were analysed using ln (1 + intake)-transformed variables. Chocolate intake was included in the composite EDNP dietary score and in descriptive analyses, but was not modelled as a separate adjusted dietary outcome because the adjusted analyses focused on the overall EDNP construct and selected major EDNP indicators, while limiting redundant statistical testing.

### 2.7. Preterm Birth Outcome

The main obstetric outcome was preterm birth, defined as delivery before 37 completed weeks of gestation. Gestational age at birth was obtained from obstetric medical records. Term birth was used as the reference outcome category in logistic regression analyses.

### 2.8. Covariates

Covariates were selected a priori according to the outcome model, biological plausibility, and the need to maintain parsimonious models.

For models evaluating dietary outcomes, covariates included maternal age, pregestational BMI, parity, educational level, and household income. These variables were selected because they may influence both physical activity and dietary behaviour during pregnancy.

For models evaluating preterm birth, the prespecified obstetric adjustment set included maternal age ≥ 35 years, pregestational BMI < 18.5 or ≥25.0 kg/m^2^, parity, smoking during pregnancy, alcohol use during pregnancy, and supplement use during pregnancy. A final diet-informed preterm birth model additionally included the composite EDNP dietary score and fruit–vegetable adequacy.

Maternal age and pregestational BMI were analysed as continuous variables in dietary models. In preterm birth models, maternal age was dichotomised as <35 vs. ≥35 years, and pregestational BMI was dichotomised as 18.5–24.9 kg/m^2^ versus <18.5 or ≥25.0 kg/m^2^. Parity was analysed as primiparity versus multiparity. Smoking, alcohol use, supplement use, and fruit–vegetable adequacy were analysed as binary variables. Supplement use was recorded as a binary variable indicating any use of vitamins, minerals, or other dietary supplements during pregnancy; information on supplement type, dose, timing, and duration was not available. Therefore, supplement use was retained as a binary covariate and could not be further classified.

### 2.9. Statistical Analysis

Statistical analyses were performed using IBM SPSS Statistics version 26 (IBM Corp., Armonk, NY, USA). The main independent variable was physical activity category, with four levels defined by quartiles of total PPAQ-derived MET-hours/week: category 1, category 2, category 3, and category 4. Category 1 represented the lowest physical activity category and was used as the reference category in regression models. Continuous variables were evaluated for distributional characteristics using histograms, Q–Q plots, skewness, kurtosis, and the Shapiro–Wilk test. Continuous variables with non-normal distributions were summarised as medians and interquartile ranges. Categorical variables were summarised as frequencies and percentages.

Participant characteristics, dietary indicators, and preterm birth frequency were compared across the four physical activity categories. Kruskal–Wallis tests were used because the continuous variables compared across categories were non-normally distributed. Chi-square tests were used for categorical variables. Linear-by-linear association tests were used when ordinal trends across physical activity categories were relevant.

Adjusted associations between physical activity categories and continuous dietary outcomes were evaluated using general linear models. Outcomes included the composite EDNP dietary score, log-transformed fast-food intake, log-transformed sweets intake, log-transformed sugar-sweetened beverage intake, and log-transformed total fruit–vegetable intake. These models were adjusted for maternal age, pregestational BMI, parity, educational level, and household income. Estimated marginal means were calculated for each physical activity category, and Bonferroni-adjusted pairwise comparisons were used for post hoc contrasts. For log-transformed dietary outcomes, estimated marginal means and their 95% confidence intervals were back-transformed to the original intake scale using exp (estimate) − 1 and reported as adjusted geometric means to improve interpretability.

Logistic regression was used to evaluate the association between physical activity category and fruit–vegetable adequacy. This model was adjusted for maternal age, pregestational BMI, parity, educational level, and household income.

The association between physical activity category and preterm birth was evaluated using binary logistic regression. Three models were fitted. Model 1 included physical activity category only. Model 2 adjusted for maternal age ≥ 35 years, pregestational BMI < 18.5 or ≥25.0 kg/m^2^, parity, smoking during pregnancy, alcohol use during pregnancy, and supplement use during pregnancy. Model 3 further included the composite EDNP dietary score and fruit–vegetable adequacy. A sensitivity analysis additionally adjusted the diet-informed preterm birth model for educational level and household income to evaluate whether the association between physical activity category and preterm birth was robust to socioeconomic adjustment.

Results from logistic regression models are reported as odds ratios with 95% confidence intervals. Results from general linear models are reported as adjusted means or back-transformed adjusted geometric means with 95% confidence intervals and overall *p*-values. All statistical tests were two-sided, and *p* < 0.05 was considered statistically significant. No formal adjustment for multiple testing was applied; interpretation considered the consistency of effect direction, effect magnitude, confidence intervals, and biological plausibility.

### 2.10. Ethical Considerations

The study was conducted in accordance with the Declaration of Helsinki. Ethical approval was obtained from the Ethics Council of the Emergency County Clinical Hospital St. Ioan cel Nou Suceava, Romania (Approval No. 29; date of approval: 20 June 2024), and from the Ethics Committee for Scientific Research of the George Emil Palade University of Medicine, Pharmacy, Science, and Technology of Targu Mures, Romania (Approval No. 3295; date of approval: 8 July 2024).

All participants received information about the study aims and procedures and provided written informed consent before enrolment. Participation was voluntary, questionnaire responses were anonymous, and clinical data were coded and used exclusively for research purposes.

## 3. Results

### 3.1. Participant Characteristics According to Physical Activity Category

The final analytical sample included 1048 mother–newborn pairs. Overall, 118 births were preterm (11.3%), while 930 occurred at term (88.7%). Because physical activity categories were defined using quartiles of total PPAQ-derived MET-hours/week, each category included 262 participants. Category 1 represented the lowest physical activity level, whereas category 4 represented the highest physical activity level.

Maternal and perinatal characteristics according to physical activity category are presented in Table 1. Maternal age differed modestly across physical activity categories, with the highest median age observed in category 3. Pregestational BMI also differed across categories, with higher median values in categories 3 and 4. Multiparity increased progressively across physical activity categories. Higher educational level and higher household income were less frequent in category 4 than in the lower physical activity categories. Smoking, alcohol use, and supplement use during pregnancy did not differ significantly across physical activity categories.

Gestational age at birth did not differ significantly across physical activity categories. However, birth weight varied across categories, with the highest median birth weight observed in category 3. The proportion of preterm birth differed significantly across physical activity categories, decreasing from 16.0% in category 1 to 6.9% in category 3, followed by a moderate increase to 10.3% in category 4.

### 3.2. Dietary Profile According to Physical Activity Category

Dietary indicators according to physical activity category are presented in Table 2. The composite EDNP dietary score differed significantly across physical activity categories, with progressively higher median values from category 1 to category 4. A similar pattern was observed for the individual EDNP components. Median intake of fast food, sweets, chocolate, and sugar-sweetened beverages was highest in the highest physical activity category.

Total fruit–vegetable intake also differed significantly across physical activity categories, increasing from a median of 553.6 g/day in category 1 to 767.2 g/day in category 4. The proportion of women meeting individualised fruit–vegetable recommendations increased from 39.7% in categories 1 and 2 to 56.9% in category 4. Overall, the descriptive dietary profile was mixed: higher physical activity categories were characterised by greater fruit–vegetable intake and adequacy, but also by higher EDNP intake.

### 3.3. Adjusted Associations Between Physical Activity Category and Dietary Outcomes

After adjustment for maternal age, pregestational BMI, parity, educational level, and household income, physical activity category remained significantly associated with the composite EDNP dietary score and with all modelled dietary outcomes (Table 3). Adjusted EDNP scores increased across physical activity categories, with the highest value observed in category 4. In Bonferroni-adjusted pairwise comparisons, category 4 had significantly higher EDNP scores than all lower categories, while category 3 also differed significantly from category 1.

Physical activity category was also associated with fast-food intake, sweets intake, sugar-sweetened beverage intake, and total fruit–vegetable intake in adjusted models. Category 4 consistently showed the highest adjusted geometric means. Pairwise comparisons indicated that category 4 differed significantly from category 1 for fast-food intake, from categories 1 and 2 for sweets intake, from all lower categories for sugar-sweetened beverage intake, and from categories 1 and 2 for total fruit–vegetable intake.

In the adjusted logistic regression model, physical activity category remained associated with fruit–vegetable adequacy. Compared with category 1, the odds of meeting individualised fruit–vegetable recommendations were higher in category 3 and category 4, with the strongest association observed for category 4.

### 3.4. Physical Activity Category and Preterm Birth

The association between physical activity category and preterm birth is shown in Table 4. In the unadjusted model, physical activity category was significantly associated with preterm birth overall. Compared with category 1, category 3 was associated with lower odds of preterm birth, while category 2 showed a non-significant reduction and category 4 showed a borderline association.

After adjustment for maternal age ≥ 35 years, pregestational BMI < 18.5 or ≥25.0 kg/m^2^, parity, smoking during pregnancy, alcohol use during pregnancy, and supplement use during pregnancy, the overall association between physical activity category and preterm birth remained statistically significant. Category 3 remained associated with lower odds of preterm birth compared with category 1.

In the diet-informed model, category 3 remained associated with lower odds of preterm birth, while category 4 also reached statistical significance. The lowest odds were consistently observed in category 3, suggesting that the association between total pregnancy physical activity and preterm birth was not strictly linear.

In a sensitivity model additionally adjusted for educational level and household income, the pattern of association between physical activity category and preterm birth was materially unchanged. Compared with category 1, category 3 remained associated with the lowest odds of preterm birth (OR 0.38, 95% CI 0.21–0.70; *p* = 0.002), while category 4 also remained associated with lower odds (OR 0.55, 95% CI 0.32–0.97; *p* = 0.038); the overall association for physical activity category remained significant (*p* = 0.014).

## 4. Discussion

The present study examined the associations between total physical activity during pregnancy, individualised fruit–vegetable adequacy, EDNP food and beverage intake, and preterm birth in a sample of 1048 postpartum women. Three main findings emerged. First, higher physical activity categories were associated with a mixed dietary profile, characterised by greater fruit–vegetable intake and higher probability of meeting individualised fruit–vegetable recommendations, but also by higher EDNP intake. Second, these associations remained evident after adjustment for maternal age, pregestational BMI, parity, educational level, and household income. Third, the lowest odds of preterm birth were observed in the third physical activity category across the logistic models, while the overall pattern did not suggest a strictly linear dose–response association. This association remained evident after adjustment for maternal, behavioural, and dietary factors. Overall, the findings support the working hypothesis that physical activity categories are associated with both dietary adequacy and EDNP intake, while also indicating that total pregnancy physical activity should not be interpreted as a simple marker of a uniformly favourable lifestyle pattern. However, these findings should be interpreted within the context of the present study population and design.

The association between higher physical activity and greater fruit–vegetable intake is broadly consistent with evidence indicating that healthier dietary patterns during pregnancy are associated with more favourable maternal and neonatal outcomes [3,4]. Fruit and vegetable intake is commonly used as a marker of dietary adequacy in pregnancy because these foods provide fibre, micronutrients, vitamin C, polyphenols, and other bioactive compounds that are relevant to maternal metabolic adaptation, oxidative balance, placental function, and fetal growth [2,15]. Previous studies have associated higher fruit and vegetable intake or plant-rich dietary patterns with fetal and infant growth indicators [16], lower odds of low birth weight in some cohorts [17], placental markers [18], and lower preeclampsia risk [19]. In the present study, the use of an individualised adequacy indicator provides an additional layer of interpretation, because fruit–vegetable adequacy was evaluated relative to estimated energy requirements rather than using a single absolute intake threshold. This approach is relevant because pregnancy energy requirements vary according to maternal characteristics, gestational needs, and assumed activity level [14,15]. The higher odds of meeting fruit–vegetable recommendations among women in the upper physical activity categories may reflect clustering of health-promoting behaviours rather than a direct effect of physical activity on diet. In pregnancy cohorts, women with higher activity or healthier lifestyle profiles tend to show better adherence to dietary guidance, including fruit and vegetable recommendations, although overall compliance with pregnancy dietary guidelines remains low [20,21,22,23]. This association may also partly reflect higher total energy intake or unmeasured differences in health awareness among more active women, both of which could increase the likelihood of consuming sufficient absolute servings of fruits and vegetables [24,25].

A central finding of this study, however, is that higher physical activity was also associated with higher EDNP intake. This included higher adjusted intake of fast food, sweets, and sugar-sweetened beverages, while the descriptive analyses also showed higher chocolate intake in the highest physical activity category. This pattern challenges the assumption that women with higher physical activity necessarily have an overall healthier dietary profile. EDNP foods and beverages are relevant during pregnancy because they may contribute to excess energy intake, poorer overall diet quality, higher glycaemic load, gestational weight gain, insulin resistance, and inflammatory or oxidative pathways potentially linked to adverse pregnancy outcomes [5,6,26]. Nevertheless, in the present sample, higher EDNP intake did not occur in isolation. It coexisted with higher fruit–vegetable intake and higher fruit–vegetable adequacy, a pattern compatible with higher overall reported consumption rather than a simple contrast between healthy and unhealthy eating behaviours. However, because total energy intake was not included as a modelled dietary outcome, this interpretation should remain cautious.

This mixed profile may be particularly important when interpreting total physical activity assessed using the PPAQ. Unlike interventions focused on structured exercise, the PPAQ captures multiple domains, including household, caregiving, transportation, occupational, sports/exercise, and sedentary activities [10]. MET-based scoring also reflects estimated energy expenditure across heterogeneous activities rather than one uniform behavioural construct [11]. Consequently, higher total MET-hours/week may represent several distinct realities: intentional exercise, greater household and caregiving workload, physically demanding occupational activity, increased mobility, or combinations of these domains. In this context, higher EDNP intake among more active women may reflect higher energy requirements, appetite-related responses to activity, time constraints, convenience-oriented food choices, or other unmeasured behavioural factors. Previous work supports a potential relationship between physical activity and food intake regulation [27], while fast-food consumption during pregnancy has been linked to poorer dietary quality in vulnerable populations [28]. Because the present study did not assess reasons for food choice, appetite, or post-activity eating, these explanations should be considered hypothesis-generating.

The results also contribute to the literature on physical activity and preterm birth. Current clinical and public health recommendations support regular physical activity during uncomplicated pregnancy, and available evidence indicates that prenatal exercise is not associated with an increased risk of preterm birth in women without contraindications [7,8,9]. The present findings are compatible with this safety profile: higher physical activity categories were not associated with increased odds of preterm birth. However, these observational findings should not be interpreted as evidence that a specific physical activity category prevents preterm birth. Instead, the lowest odds were observed in the third physical activity category, and this association remained stable across unadjusted, obstetrically adjusted, and diet-informed models. This persistence after additional adjustment for EDNP score and fruit–vegetable adequacy suggests that the association between physical activity category and preterm birth was not explained solely by the two dietary dimensions included in the final model.

The non-linear pattern observed for preterm birth is noteworthy. Category 3 showed the most consistent inverse association with preterm birth. Category 4 had odds ratios below 1.00 in all three models, but its confidence interval excluded 1.00 only in the diet-informed model. This pattern does not support a simple dose–response interpretation in which progressively higher total physical activity is uniformly associated with progressively lower odds of preterm birth. Similar domain-dependent associations have been reported in pregnancy, with leisure-time physical activity showing more consistently favourable associations than domestic, commuting, or occupationally related activity [29]. This may suggest that the highest total activity category captured not only structured exercise but also household, caregiving, and occupational workload, domains whose health implications may differ from leisure-time exercise and may be shaped by social and employment-related constraints [30,31].

Several biological and behavioural pathways may provide plausibility for the observed association, although they were not directly measured in this study. Physical activity during pregnancy has been linked to improved metabolic regulation, lower excessive gestational weight gain, reduced inflammatory burden, and better psychological well-being [7,32]. These pathways overlap with mechanisms that have been implicated in preterm birth, including inflammatory, vascular, metabolic, and stress-related neuroendocrine processes [33,34]. However, because this study was observational and relied on postpartum exposure assessment, these mechanisms remain hypothetical and cannot be directly tested. In addition, preterm birth is a heterogeneous outcome comprising distinct subtypes, including spontaneous preterm labour, preterm premature rupture of membranes, and clinician-initiated preterm delivery, which may have different determinants [35,36]. This distinction is important because the same physical activity or dietary exposure may not have identical associations across preterm birth subtypes.

An alternative explanation that should be considered is reverse causality. Because physical activity was assessed retrospectively after delivery, women who experienced symptoms suggestive of threatened preterm birth or who were advised to restrict activity during pregnancy may have reported lower levels of physical activity [7]. Consequently, lower physical activity could partly reflect underlying pregnancy complications or perceived risk rather than an antecedent behavioural exposure. Although the observed association remained after adjustment for several maternal and dietary factors, the cross-sectional design and retrospective exposure assessment preclude establishing temporal directionality. Therefore, the findings should be interpreted as associations rather than evidence of a protective effect of physical activity on preterm birth.

The coexistence of higher fruit–vegetable adequacy and higher EDNP intake also has implications for prenatal counselling. In clinical practice, counselling often addresses physical activity and diet as separate behaviours, yet the present findings suggest that they should be considered jointly. In this sample, women in higher physical activity categories were more likely to meet fruit–vegetable recommendations but also reported higher EDNP intake. These findings suggest that dietary assessment should not be omitted solely because a pregnant woman reports higher activity levels. Conversely, women in lower physical activity categories may require both safe movement-oriented counselling and support for achieving adequate fruit–vegetable intake. Therefore, prenatal lifestyle guidance should avoid assuming that favourable behaviour in one domain implies favourable behaviour in all domains. Integrated antenatal counselling that combines safe physical activity promotion with guidance on discretionary foods, sugar-sweetened beverages, and overall dietary quality may be more appropriate than single-behaviour advice [7,23,37].

These findings are relevant from a nutritional epidemiology perspective, because pregnancy behaviours such as diet quality, physical activity, adiposity, and sociodemographic characteristics may cluster rather than operate as isolated exposures [38]. The present results show that physical activity categories can be associated with apparently favourable and unfavourable dietary indicators at the same time. This supports the need for analytical approaches that consider multiple lifestyle dimensions simultaneously, rather than classifying participants as broadly “healthy” or “unhealthy” on the basis of a single exposure. Future studies could use dietary pattern analysis, latent class analysis, or lifestyle pattern clustering to identify subgroups of pregnant women characterised by distinct combinations of activity, diet quality, EDNP intake, sleep, stress, and socioeconomic context [39]. Such approaches may provide a more realistic representation of maternal lifestyle during pregnancy.

The study has several strengths. It included a relatively large sample of postpartum women recruited consecutively in a tertiary maternity setting. Preterm birth and gestational age were obtained from obstetric medical records, reducing outcome misclassification compared with maternal recall alone. Physical activity was assessed using a pregnancy-specific questionnaire, and dietary intake was evaluated using an adapted food frequency questionnaire. The analysis also combined descriptive dietary profiles with adjusted models, allowing the evaluation of both fruit–vegetable adequacy and EDNP intake across physical activity categories. In addition, the main fruit–vegetable adequacy analysis used a fixed low-active coefficient to reduce circularity between the physical activity exposure and the adequacy outcome.

Several limitations should be acknowledged. First, the cross-sectional postpartum design prevents causal inference. Dietary intake and physical activity were assessed retrospectively after delivery, and recall may have been influenced by pregnancy outcome, postpartum circumstances, or social desirability. The exclusion of women with maternal diabetes and neonates with macrosomia may also limit generalisability to pregnancies with altered metabolic risk profiles. Second, both diet and physical activity were self-reported, which may have introduced measurement error and misclassification. Food frequency questionnaires are useful for ranking participants according to usual intake, but estimates of absolute intake may be less precise, particularly for specific foods or beverages consumed episodically, such as sweets, fast food, and sugar-sweetened beverages [40]. Similarly, questionnaire-based physical activity assessment during pregnancy may be affected by recall and classification error and may perform less accurately than objective monitoring methods such as accelerometry [41]. Although the dietary questionnaire and physical activity assessment were administered in Romanian, formal validation of the resulting assessment package in this specific population was not performed. Third, the physical activity categories were based on quartiles of the sample distribution rather than externally validated pregnancy-specific thresholds; therefore, they should be interpreted as relative exposure categories within this sample and should not be translated into clinical activity cut-offs. Fourth, the EDNP score was based on selected food and beverage groups available in the questionnaire and did not capture the full complexity of ultra-processed food intake, total energy intake, macronutrient distribution, or overall dietary quality. Additional EDNP or ultra-processed foods, such as processed snacks, refined pastries, processed meat products, sauces, and other discretionary foods, were not comprehensively assessed as a unified category and therefore could not be incorporated into the composite score. Fifth, although preterm birth was obtained from medical records, spontaneous and medically indicated preterm births were not analysed separately.

Finally, residual confounding and reverse causation remain possible despite adjustment for relevant maternal, behavioural, socioeconomic, and dietary factors. Supplement use was assessed only as a binary variable, without information on supplement type, dose, timing, or duration; therefore, residual confounding related to specific supplements cannot be excluded. Women with symptoms, medical advice to restrict activity, or early pregnancy complications may have reported lower activity, which could partly influence the observed association with preterm birth.

Future research should examine these associations prospectively, ideally with repeated assessments of diet and physical activity across pregnancy. Studies combining pregnancy-specific questionnaires with accelerometry would help distinguish total activity volume from intensity, sedentary time, and domain-specific activity. Further analyses should separate leisure-time exercise from occupational, household, and caregiving activity, because these domains may have different associations with dietary behaviour and obstetric outcomes [29,42]. Future studies should also distinguish spontaneous from medically indicated preterm birth and examine metabolic, inflammatory, gestational-weight-gain, and placental pathways as potential mediators or effect modifiers [43,44]. These approaches would help clarify whether the non-linear pattern observed in this study reflects differences in physical activity domains, reverse causation, residual socioeconomic and clinical confounding, or potential physiological benefit at intermediate-to-high activity levels.

## 5. Conclusions

In this sample of postpartum women, total physical activity during pregnancy was associated with a mixed dietary profile. Higher physical activity categories were characterised by greater fruit–vegetable intake and higher odds of meeting individualised fruit–vegetable recommendations, but also by higher EDNP food and beverage intake. These findings suggest that total pregnancy physical activity should not necessarily be interpreted as a marker of a uniformly favourable lifestyle profile.

Physical activity category was also associated with preterm birth, with the lowest odds observed in the third activity category. This pattern suggests a non-linear association rather than a simple dose–response relationship between progressively higher total physical activity and lower odds of preterm birth. Because of the observational design, postpartum exposure assessment, and the possibility of reverse causality, these findings should be interpreted as associations rather than causal effects.

The results support an integrated approach to lifestyle assessment during pregnancy, in which physical activity and diet are considered together. Particular attention to EDNP foods and sugar-sweetened beverages may be warranted when counselling pregnant women reporting higher physical activity levels, as greater physical activity in this sample coexisted with higher intake of these dietary components despite higher fruit–vegetable adequacy. Prospective studies with repeated dietary and physical activity assessments, domain-specific activity measures, and differentiation between spontaneous and medically indicated preterm birth are needed to clarify the temporal and mechanistic pathways linking maternal lifestyle profiles with preterm birth.

## Figures and Tables

**Table 1:** Maternal and perinatal characteristics according to physical activity category (*n* = 1048).

Characteristic	Physical Activity Category				*p*-Value
	Category 1 *n* = 262	Category 2 *n* = 262	Category 3 *n* = 262	Category 4 *n* = 262	
Maternal age, years	28.0 (24.0–33.0)	28.0 (25.0–33.0)	29.0 (26.0–33.0)	28.0 (24.0–32.0)	0.024
Maternal age ≥ 35 years	46 (17.6)	42 (16.0)	51 (19.5)	33 (12.6)	0.184
Pregestational BMI, kg/m^2^	23.10 (20.87–25.89)	23.21 (20.93–26.98)	24.39 (21.80–27.21)	24.14 (21.40–28.06)	0.002
Pregestational BMI < 18.5 or ≥25.0 kg/m^2^	94 (35.9)	115 (43.9)	122 (46.6)	122 (46.6)	0.042
Multiparity	112 (42.7)	164 (62.6)	190 (72.5)	205 (78.2)	<0.001
Education > 12 years	106 (40.5)	108 (41.2)	106 (40.5)	79 (30.2)	0.025
Higher household income	156 (59.5)	147 (56.1)	126 (48.1)	121 (46.2)	0.005
Smoking during pregnancy	74 (28.2)	75 (28.6)	72 (27.5)	84 (32.1)	0.667
Alcohol use during pregnancy	31 (11.8)	40 (15.3)	43 (16.4)	49 (18.7)	0.179
Supplement use during pregnancy	224 (85.5)	223 (85.1)	219 (83.6)	209 (79.8)	0.273
Gestational age at birth, weeks	39.0 (38.0–39.0)	39.0 (38.0–39.0)	39.0 (38.0–39.0)	39.0 (38.0–39.0)	0.244
Birth weight, g	3250 (2900–3520)	3255 (2900–3555)	3395 (3025–3650)	3200 (2900–3543)	0.009
Preterm birth	42 (16.0)	31 (11.8)	18 (6.9)	27 (10.3)	0.010

Note: Data are presented as median (interquartile range) for continuous variables and *n* (%) for categorical variables. Physical activity categories were based on quartiles of total PPAQ-derived MET-hours/week. *p*-values were obtained using Kruskal–Wallis tests for continuous variables and Pearson chi-square tests for categorical variables. BMI, body mass index; PPAQ, Pregnancy Physical Activity Questionnaire; MET, metabolic equivalent of task.

**Table 2:** Dietary profile according to physical activity category (*n* = 1048).

Dietary Indicator	Physical Activity Category				*p*-Value
	Category 1 *n* = 262	Category 2 *n* = 262	Category 3 *n* = 262	Category 4 *n* = 262	
Composite EDNP dietary score	−0.26 (−0.94–0.45)	−0.17 (−0.72–0.55)	−0.02 (−0.60–0.67)	0.43 (−0.41–1.11)	<0.001
Fast-food intake, g/day	46.1 (24.4–94.6)	55.2 (31.4–102.5)	56.1 (32.4–97.2)	68.6 (30.9–126.9)	0.002
Sweets intake, g/day	67.4 (34.7–149.5)	79.0 (41.5–150.5)	75.5 (41.9–165.0)	129.8 (61.4–277.2)	<0.001
Chocolate intake, g/day	1.4 (0.0–9.0)	1.7 (0.0–9.0)	4.0 (0.0–15.8)	6.3 (0.0–33.0)	<0.001
Sugar-sweetened beverages, mL/day	87.4 (32.0–257.0)	102.9 (44.0–299.4)	120.9 (36.0–360.0)	222.9 (60.0–689.6)	<0.001
Total fruit–vegetable intake, g/day	553.6 (321.6–948.4)	533.0 (321.9–955.1)	618.7 (355.1–1172.0)	767.2 (452.1–1338.1)	<0.001
Adequate fruit–vegetable intake, *n* (%)	104 (39.7)	104 (39.7)	122 (46.6)	149 (56.9)	<0.001

Note: Data are presented as median (interquartile range) for continuous variables and *n* (%) for categorical variables. Physical activity categories were based on quartiles of total PPAQ-derived MET-hours/week. *p*-values were obtained using Kruskal–Wallis tests for continuous variables and Pearson chi-square tests for fruit–vegetable adequacy. EDNP, energy-dense, nutrient-poor; PPAQ, Pregnancy Physical Activity Questionnaire; MET, metabolic equivalent of task.

**Table 3:** Adjusted associations between physical activity category and dietary outcomes (*n* = 1048).

Dietary Outcome	Physical Activity Category				Overall *p*-Value
	Category 1 *n* = 262	Category 2 *n* = 262	Category 3 *n* = 262	Category 4 *n* = 262	
Composite EDNP dietary score, adjusted mean	−0.250 (−0.367 to −0.133)	−0.087 (−0.204 to 0.030)	0.024 (−0.095 to 0.143)	0.270 (0.147 to 0.393)	<0.001
Fast-food intake, adjusted geometric mean, g/day	46.4 (41.2 to 52.3)	57.6 (51.1 to 64.8)	56.3 (49.9 to 63.5)	63.8 (56.3 to 72.3)	0.003
Sweets intake, adjusted geometric mean, g/day	67.6 (58.7 to 77.7)	78.8 (68.5 to 90.7)	84.9 (73.7 to 97.9)	107.3 (92.6 to 124.3)	<0.001
Sugar-sweetened beverage intake, adjusted geometric mean, mL/day	80.5 (65.6 to 98.6)	96.7 (78.9 to 118.5)	99.6 (81.1 to 122.2)	155.6 (125.8 to 192.4)	<0.001
Total fruit–vegetable intake, adjusted geometric mean, g/day	522.2 (470.1 to 580.1)	554.0 (498.7 to 615.5)	634.9 (570.9 to 706.0)	752.7 (674.2 to 841.2)	<0.001
Fruit–vegetable adequacy, OR (95% CI)	Reference	1.07 (0.75 to 1.53)	1.48 (1.03 to 2.12)	2.24 (1.55 to 3.24)	<0.001

Note: Values for the composite EDNP dietary score are adjusted means with 95% confidence intervals on the standardised score scale. Values for fast-food intake, sweets intake, sugar-sweetened beverage intake, and total fruit–vegetable intake are back-transformed adjusted geometric means with 95% confidence intervals; models were fitted using ln (1 + intake)-transformed variables, and estimates were back-transformed as exp (estimate) − 1. The model for fruit–vegetable adequacy was fitted using logistic regression and is presented as odds ratios with 95% confidence intervals, with category 1 as the reference group. All models were adjusted for maternal age, pregestational BMI, parity, educational level, and household income. EDNP, energy-dense, nutrient-poor; OR, odds ratio; CI, confidence interval.

**Table 4:** Associations between physical activity category and preterm birth (*n* = 1048).

Physical Activity Category	Model 1 OR (95% CI)	*p*-Value	Model 2 OR (95% CI)	*p*-Value	Model 3 OR (95% CI)	*p*-Value
Category 1	Reference	—	Reference	—	Reference	—
Category 2	0.70 (0.43–1.16)	0.167	0.71 (0.43–1.19)	0.190	0.71 (0.42–1.17)	0.180
Category 3	0.39 (0.22–0.69)	0.001	0.39 (0.22–0.71)	0.002	0.38 (0.21–0.68)	0.001
Category 4	0.60 (0.36–1.01)	0.054	0.62 (0.36–1.06)	0.081	0.56 (0.32–0.99)	0.044
Overall effect of physical activity category	—	0.012	—	0.020	—	0.012

Note: Model 1 was unadjusted. Model 2 was adjusted for maternal age ≥ 35 years, pregestational BMI < 18.5 or ≥25.0 kg/m^2^, parity, smoking during pregnancy, alcohol use during pregnancy, and supplement use during pregnancy. Model 3 was additionally adjusted for the composite EDNP dietary score and fruit–vegetable adequacy. Category 1 was the reference group. Dashes (—) indicate values that are not applicable; no p-value was calculated for the reference category, and no odds ratio is reported for the overall effect test. OR, odds ratio; CI, confidence interval; EDNP, energy-dense, nutrient-poor.

## Data Availability

The data presented in this study are available on request from the corresponding author due to privacy and ethical restrictions related to participant-level health data.

## Abbreviations

The following abbreviations are used in this manuscript:

BMI	Body mass index
CI	Confidence interval
EDNP	Energy-dense, nutrient-poor
MET	Metabolic equivalent of task
OR	Odds ratio
PPAQ	Pregnancy Physical Activity Questionnaire
